# Broadband Photoreceptors Are Involved in Violet Light Preference in the Parasitoid Fly *Exorista Japonica*

**DOI:** 10.1371/journal.pone.0160441

**Published:** 2016-08-17

**Authors:** Yoshiaki Tokushima, Takuya Uehara, Terumi Yamaguchi, Kentaro Arikawa, Yooichi Kainoh, Masami Shimoda

**Affiliations:** 1 Institute of Agrobilogical Science, NARO, Tsukuba, Ibaraki, Japan; 2 Graduate School of Life and Environmental Sciences, University of Tsukuba, Ibaraki, Japan; 3 Laboratory of Neuroethology, SOKENDAI (The Graduate University for Advanced Studies), Shonan Village, Hayama, Japan; 4 Faculty of Life and Environmental Sciences, University of Tsukuba, Ibaraki, Japan; Tohoku University, JAPAN

## Abstract

Phototaxis has been described in many insects, which are often attracted to specific wavelengths of light. However, little is known about phototaxis in parasitoid insect species that are potentially useful for integrated pest management. In this study, we investigated the wavelength dependency of the phototactic behavior of the parasitoid fly *Exorista japonica* and its possible mechanism. Multiple-choice tests with six monochromatic stimuli revealed that the flies were specifically attracted to violet light peaking at 405 nm, which was unexpected because insects are generally attracted to ultraviolet or green light. We measured the spectral sensitivity of the compound eye, and found that the sensitivity peaked at 340 nm, as in other brachyceran flies. We used statistical modeling and optimization of the process parameters to predict the type of photoreceptor contributing to the violet preference. The analysis revealed that the wavelength preference could be explained by linear models of the quanta received by photoreceptors, including the R1-6 broadband receptors. The broadband receptors appear to contribute positively, whereas the R7-8 narrowband receptors contribute negatively to achieve the violet preference; *i*.*e*., spectral opponency might be involved.

## Introduction

Phototaxis, which is a locomotor response toward or away from light sources, is a well-characterized behavior in insects [1]. Preferred light wavelengths in phototaxis vary among insect species. For example, the fruit fly *Drosophila melanogaster* [2, 3], tsetse fly *Glossina morsitans* [4], and housefly *Musca domestica* [5] are attracted mainly to ultraviolet light (UV; wavelength = 300–380 nm), while the whitefly *Trialeurodes vaporariorum* [6] and thrip *Scirtothrips dorsalis* [7, 8] prefer green light (wavelength = 500–570 nm). UV light can be exploited during migration and spatial orientation in the landscape, because it is related to the recognition of open sky or polarized light [9]. Green is the major component of sunlight reflected by plant leaves. Therefore, green could be used for landing and foraging on foliage [10].

The compound eyes of insects are composed of a number of small units called ommatidia, with each unit containing several photoreceptor cells. The photoreceptors in a single ommatidium vary in spectral sensitivities. For example, the ommatidia of brachyceran flies bear eight photoreceptors, of which six are peripheral photoreceptors (R1-6) and two central photoreceptors (R7, R8). The R1-6 exhibit a broadband sensitivity, peaking both in the UV and blue wavelength regions. The spectral sensitivities of R7 and R8 differ among ommatidia: 350 nm-peaking R7 is always associated with 540 nm-peaking R8 in a subset of ommatidia called *yellow*-type ommatidia, while 355 nm-peaking R7 always coexists with 460 nm-peaking R8 in the *pale*-type ommatidia. These two types of ommatidia are distributed randomly in the compound eye [11]. The photoreceptors send axons into the optic lobe, where the photoreceptor signals are processed by higher-order neurons and eventually sent to the central brain. The spectrally homogeneous peripheral photoreceptor system appears to be best for motion or shape vision, while the spectral heterogeneity of central photoreceptors suggests that they are indispensable for color visualization [12].

Vision of dipteran species has been extensively studied because of their importance in hygiene management. The housefly and fruit fly are sanitary insects, while the tsetse fly is a vector of African trypanosomiasis. However, little is known about parasitoid flies, which are important for biological control in agriculture [13]. Parasitoid flies of the Tachinidae family are natural enemies, mainly attacking larvae of moths and butterflies. The female tachinid flies lay eggs on host caterpillars, and the hatched larvae eat the host tissue, causing death of the host. Newly emerged female flies visit flowers to find food, mate with males, and search for hosts to lay eggs. When searching for a host, they rely on visual cues, including host movement [14, 15], in addition to the olfactory cues from plants [16, 17].

Light illumination, using light-emitting diodes (LEDs), is becoming a powerful and energy-saving tool for integrated pest management (IPM; [18]). We have begun studies on vision in the parasitoid fly *Exorista japonica* Townsend, and in this study, we measured the wavelength preference in phototactic behavior and the compound eye spectral sensitivity. We also attempted to identify the photoreceptors contributing to wavelength preference using a statistical model.

## Materials and Methods

### Insects

The parasitoid fly *E*. *japonica* was maintained using final instar larvae of the Northern armyworm, *Mythimna separata* Walker as the host. The host caterpillars were reared on a Silk Mate 2M diet (Nosan Corporation, Kanagawa, Japan), as described previously [19]. After growing inside the host, the fly maggots emerge from the host and eventually become puparia. The flies that emerged from puparia of more than 50 mg were kept individually in plastic containers (10 cm diameter, 4 cm high) for the experiments. They were fed sugar and water [20,21]. Flies were stored individually in a plastic container after emergence, and these flies represented the unmated males or females. To obtain mated flies, one female and at least five males were placed in a container and allowed to mate. The pair that continued mating for longer than 1 h was selected as the mated male and female. All flies were used for the experiments within 6 days of emergence. All rearing and experiments were conducted at 25°C under a 16 h light– 8 h dark photoperiod.

### Wavelength choice test

**Fig 1:**
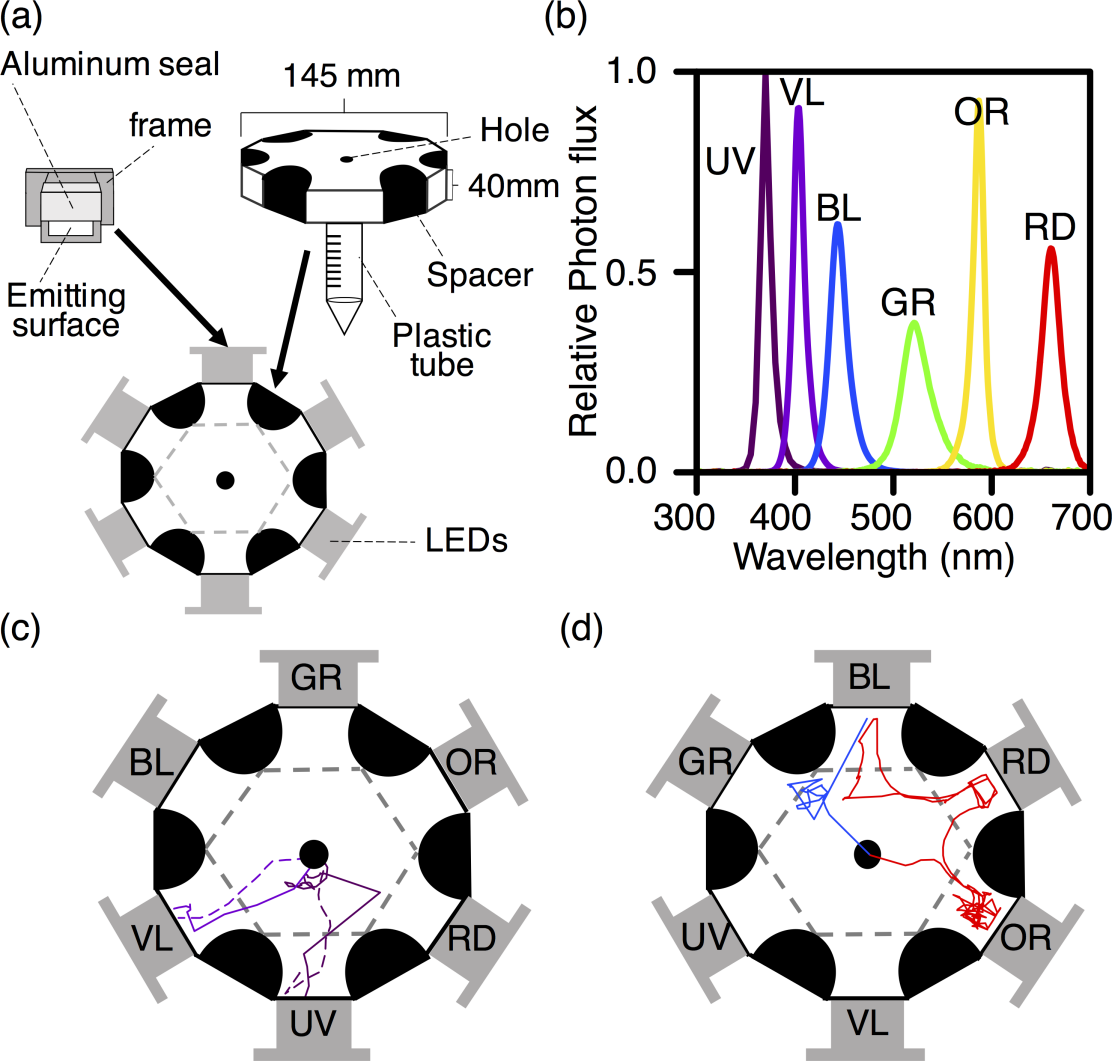
Setup for the wavelength choice test. (a) The experimental arena used for the wavelength choice test (modified from [22]). Two dodecagonal clear plastic plates (top and bottom) were separated by six semicircular black spacers. A piece of filter paper was placed on the bottom plate. A hole was made in the center, through which the fly could climb into the experimental arena via the plastic tube. Six LED lights of different colors were placed on the open sides of the arena. We used UV (365 nm), VL (405 nm), BL (450 nm), GR (525 nm), OR (590 nm), and RD (660 nm) LEDs. The light-emitting surface was made of an optical diffusion filter framed with an aluminum seal. Dashed lines indicate imaginary borders, separating each LED area from the central hexagonal area. (b) Irradiance spectra of the LEDs. The integral of the spectra was adjusted so that the photon fluxes were equivalent for all six LEDs. (c, d) Example trajectories of the flies. Several trials were merged in each Fig The color of a trajectory reflects the peak wavelength of the LED that was eventually chosen by the individual. (c) Example trajectories of the flies that chose UV or VL. (d) Example trajectories of the flies that chose LEDs other than UV or VL.

We conducted wavelength choice tests in a 12-sided polygon arena (Fig 1A), modified from Ogino et al [22]. The arena was fixed in a light-tight box, which was illuminated by infrared light. The floor of the arena was covered with filter paper, which was replaced between all trials to eliminate any possible effects of the chemical cues from the previously tested flies. Six LEDs (LDF 26 series; CCS, Kyoto, Japan) were located around the arena as shown in Fig 1A (S1 Dataset). The relative position of the LEDs was changed randomly in each trial, to avoid positional effects of the arena. The photon flux from each LED was measured with a spectrometer (HSU-100S; Asahi Spectra, Tokyo, Japan, Fig 1B). The irradiance spectrum of each LED peaked at 365, 405, 450, 525, 590, and 660 nm, thus hereafter, we refer to the LEDs as UV (ultraviolet), VL (violet), BL (blue), GR (green), OR (orange), and RD (red) for simplicity. For each LED, the integral of the irradiance spectrum over the wavelength range, between 300 and 700 nm, was adjusted to 0.15 μmol m^–2^ s^–1^ at a distance of 60 cm from the LED by regulating the input current. All behavioral experiments were performed at the same intensity, as follows:

∫I(λ)dλ=constant (1)

where *I*(*λ*) is the irradiance spectra of the LED (Fig 1B).

A fly was introduced into the arena through the hole via a 1-cm diameter plastic tube. Recording was started when the fly climbed into the arena, and continued for up to 7 min. If the fly crossed the imaginary border (dashed line) that separated each LED area from the central hexagonal area and stayed there for at least 30 s, we defined the behavior as a “choice” and stopped recording. A single fly was subjected to each trial just once and never reused. However, if a fly did not choose an LED within 7 min, we stopped recording, allowed the fly to rest for 1 day and retried the experiment the next day. A total of 50 flies were investigated for each condition (male/female, mated/unmated). The movement of the fly was recorded using an infrared camera (Himawari GE60; Library, Tokyo, Japan) connected to a monitor visible to the experimenter. Video sequences were analyzed using a program (Move-tr/2D 7.0; Library, Tokyo, Japan), and the trajectories were extracted [23].

### Electrophysiological experiments

The electroretinogram (ERG) was recorded in response to monochromatic stimuli to determine the spectral sensitivity of the fly compound eye [24]. The monochromatic stimuli were provided by a 500 W xenon lamp with a series of narrowband interference filters ranging from 300 to 740 nm (Asahi Spectra, FWHM 10–14 nm). The light beam was focused on the compound eye with a quartz optical fiber. The photon flux of the monochromatic stimulus was measured using a radiometer (model-470D; Sanso, Tokyo, Japan) and adjusted to a standard number of photons using an optical wedge. A fly was mounted on a plastic stage with beeswax in the recording chamber. A chloridized silver wire was inserted into the fly’s abdomen as the reference electrode. The tip of a fine glass pipette filled with tap water was positioned at the eye surface through a small amount of conductive paste. The ERG was recorded with a preamplifier (MEZ-7200; Nihon Kohden, Tokyo, Japan) connected to an AD converter (MP-150; BIOPAC, USA).

The fly was adapted to the dark for approximately 10 min in the Faraday cage after the electrodes were set in the Faraday cage. We then started the recording using monochromatic flashes 100 ms in duration at 5 s intervals. The wavelengths were first swept from short to long wavelengths, and then this was repeated in reverse. These pairs of bidirectional recordings were repeated five times, yielding ten spectral scans. The response–stimulus intensity function (*V*-log *I*) was also recorded over a 4-log unit intensity range at several wavelengths. The *V*-log *I* data were fitted by the Naka–Rushton function, *V*/*V*_max_ = *I*^*n*^/(*I*^*n*^ + *K*^*n*^), where *I* is the stimulus intensity, *V* is the response amplitude, *V*_max_ is the maximum response amplitude, *K* is the stimulus intensity eliciting 50% of *V*_max_, and *n* is the exponential slope. Finally, the spectral response was converted into the relative spectral sensitivity.

### Modeling wavelength preference

To clarify the contribution of the photoreceptors to phototaxis, we analyzed the wavelength preference based on the photoreceptor spectral sensitivities. The quantum catch of a receptor *Q* is calculated from the spectral photon flux of the stimulus light *I*(*λ*) and the spectral sensitivity *R*(*λ*) of the photoreceptor-in-question, as described in [25]:

Q=∫I(λ)R(λ)dλ. (2)

For compound-eye-based modeling, we calculated the relative quantum catch of the whole compound eye, *Q*_CE_, using the spectral sensitivity determined by the ERG, as described in [26]. For photoreceptor-based modeling, we calculated *Q*_i_ (i = R1-6, R7 and R8 of *pale*-ommatidia (R7p, R8p), R7 and R8 of *yellow*-ommatidia (R7y, R8y)) for individual photoreceptors. Because the spectral sensitivities of the *E*. *japonica* photoreceptors have not been determined, we used the *R*_i_(λ) value of a closely related species, *Musca domestica* [11], as described in [27–30]. The relationship between the quantum catch, *Q*, and choice rate, *C*, in the wavelength choice test was inferred statistically using a generalized linear model [31], according to [27, 32].

The choice frequency, *C*, was set as the dependent variable following the binomial distribution. In the compound-eye-based modeling, the linear equation for the quantum catch of the compound eye, *Q*_CE_, with the intercept was set as the linear predictor *η*:

$$\eta =b_{0}+b_{CE}Q_{CE},$$ (3)

where *b*_0_ is the intercept and *b*_1_ is the regression coefficient of *Q*_CE_. In the photoreceptor-based modeling, that of the photoreceptor *Q*_i_ for all possible combinations without the intercept was set as the linear predictor, as

$$\eta =\Sigma b_{i}Q_{i}.$$ (4)

While the value of the choice frequency, *C*, can range from 0 (for an LED that is never chosen) to 1 (for an LED that is always chosen), the linear predictor *η* can lie outside this range. Therefore, the logit function, Logit(*C*) = ln{*C*/(1-*C*)}, was set as a link function, as

Logit(C)=η. (5)

The inverse logit function was used to visualize the modeling results, as

C=1/{1+exp(–η)}. (6)

Since there were multiple variables in the photoreceptor-based modeling, model selection was conducted using Akaike’s information criterion (*AIC*) [33], the indicator of the fitness of a model in consideration of over fitting. The significance of the regression coefficient was assessed by the Wald test, in which z-statistics derived from the SEM of the coefficients were used. Then, we chose the models with the best fit consisting of the significant coefficients. All calculations were performed using the “glm” function in the statistical software R 3.2.2 [34].

## Results

### Wavelength choice test

**Fig 2:**
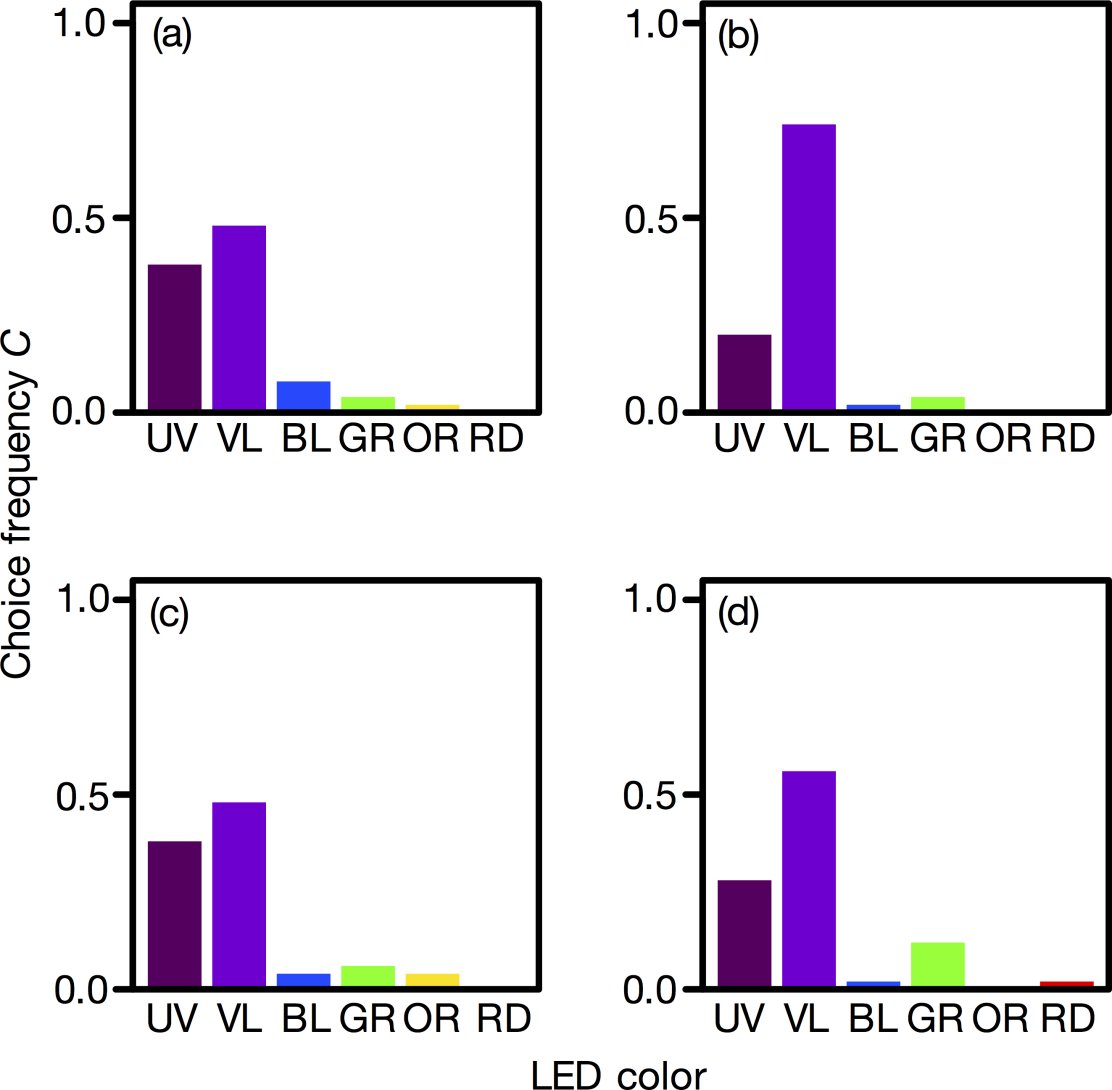
Wavelength preference of *Exorista japonica*. (a) Unmated males, (b) mated males, (c) unmated females, and (d) mated females. For each experimental group, *n* = 50.

The flies moved toward an LED with drawing trajectory, which was straight, zigzag or more complicated. The vast majority (96.5%) of flies evaluated moved straight to the LED light (Fig 1C). A few flies (3.5%) moved from one LED to another (Fig 1D) and were not observed among the flies that selected UV or VL. Fig 2 shows the LED choice frequency (S2 Dataset). Unmated flies preferred VL and UV equally (Fig 2A and 2C), while mated flies preferred VL over UV (Fig 2B and 2D). In all experimental groups, BL, GR, OR, and RD were less preferred than was UV and VL. No sexual difference in wavelength preference was detected.

### Electrophysiological experiments

**Fig 3:**
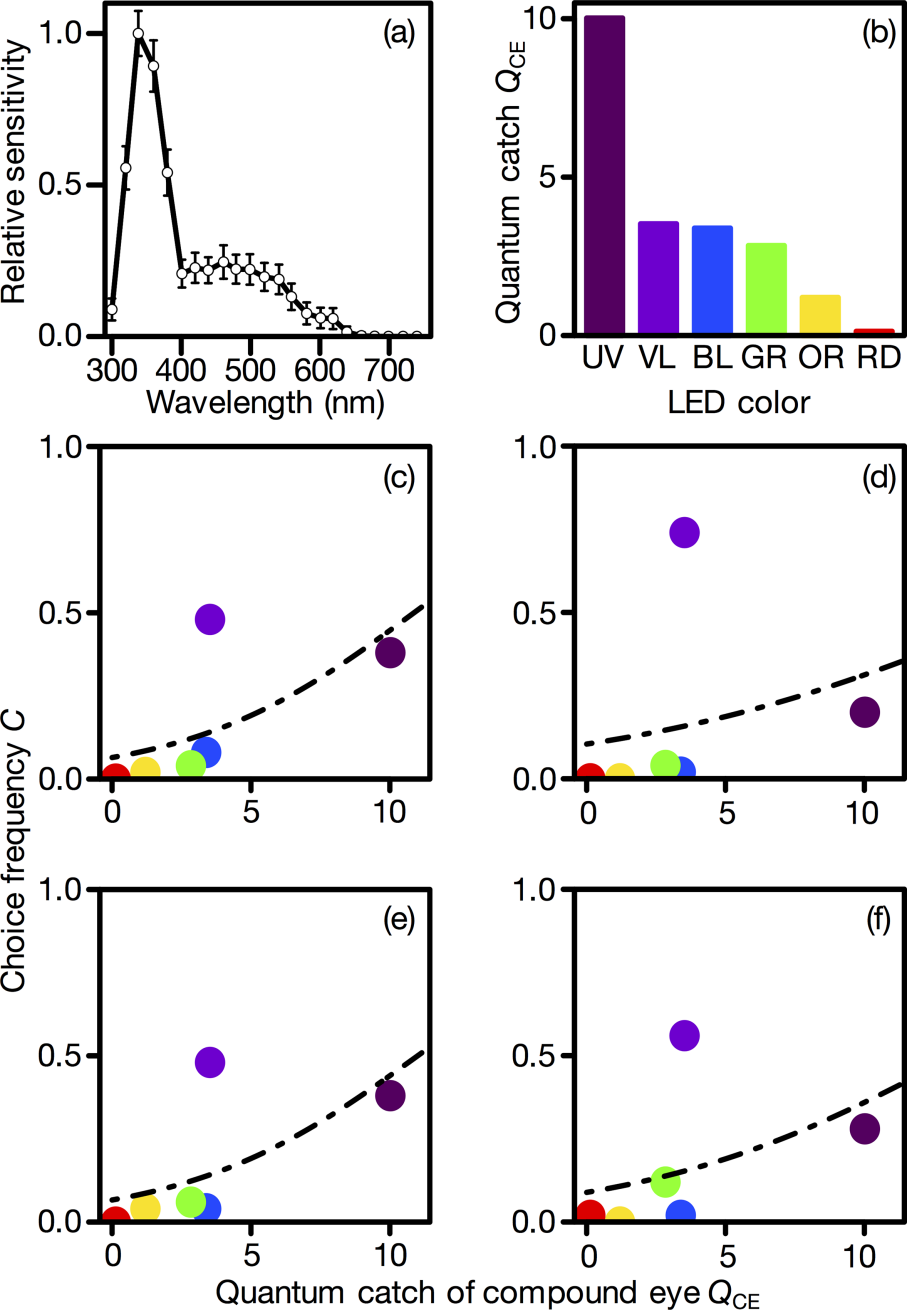
Compound-eye-based modeling of wavelength preference. (a) Spectral sensitivity of the *Exorista japonica* compound eye. Mean ± standard error. (b) The relative quantum catch of the compound eye. (c-f) The compound eye quantum catch versus the choice frequency for LEDs in (a) unmated males, (b) mated males, (c) unmated females, and (d) mated females. Circles indicate the measured choice frequency, and the dashed line is the value fitted by the model (Eq 2) as a function of *Q*_CE_. The coefficients are shown in Table 1.

We measured the spectral sensitivity of the compound eye to monochromatic light flashes in two females and three males by ERG. The spectral sensitivity exhibited a major peak around 340 nm and a secondary sensitivity band in the long wavelength region, which is typical of brachyceran flies [11]. Because no sex difference was detected, the average of all individuals is shown in Fig 3A.

### Modeling

Using the spectral sensitivity of the whole compound eye determined by ERG (Fig 3A, S1 Dataset), the quantum catch of the compound eye (*Q*_CE_) was calculated (Fig 3B). The magnitude of the quantum catch was, as expected, in parallel with the spectral sensitivity. The compound eye caught quanta mostly from the UV LED. Fig 3C–3F show the relationship between *Q*_CE_ and the choice frequency (*C*). For all experimental groups, the model (Table 1) fit the UV, BL, GR, OR, and RD LEDs. For these LEDs, the choice frequency increased with *Q*_CE_. By contrast, the model did not fit VL, the preferred LED (Fig 3C–3F).

**Table 1:** Regression coefficients in the compound-eye-based model for each experimental group.

Experimental group	*b*_0_ (intercept)	*b*_CE_
Unmated male	–2.67*	0.25*
Mated male	–2.14*	0.13*
Unmated female	–2.64*	0.24*
Mated female	–2.33*	0.18*

* *p* < 0.05 (*z*-test).

**Fig 4:**
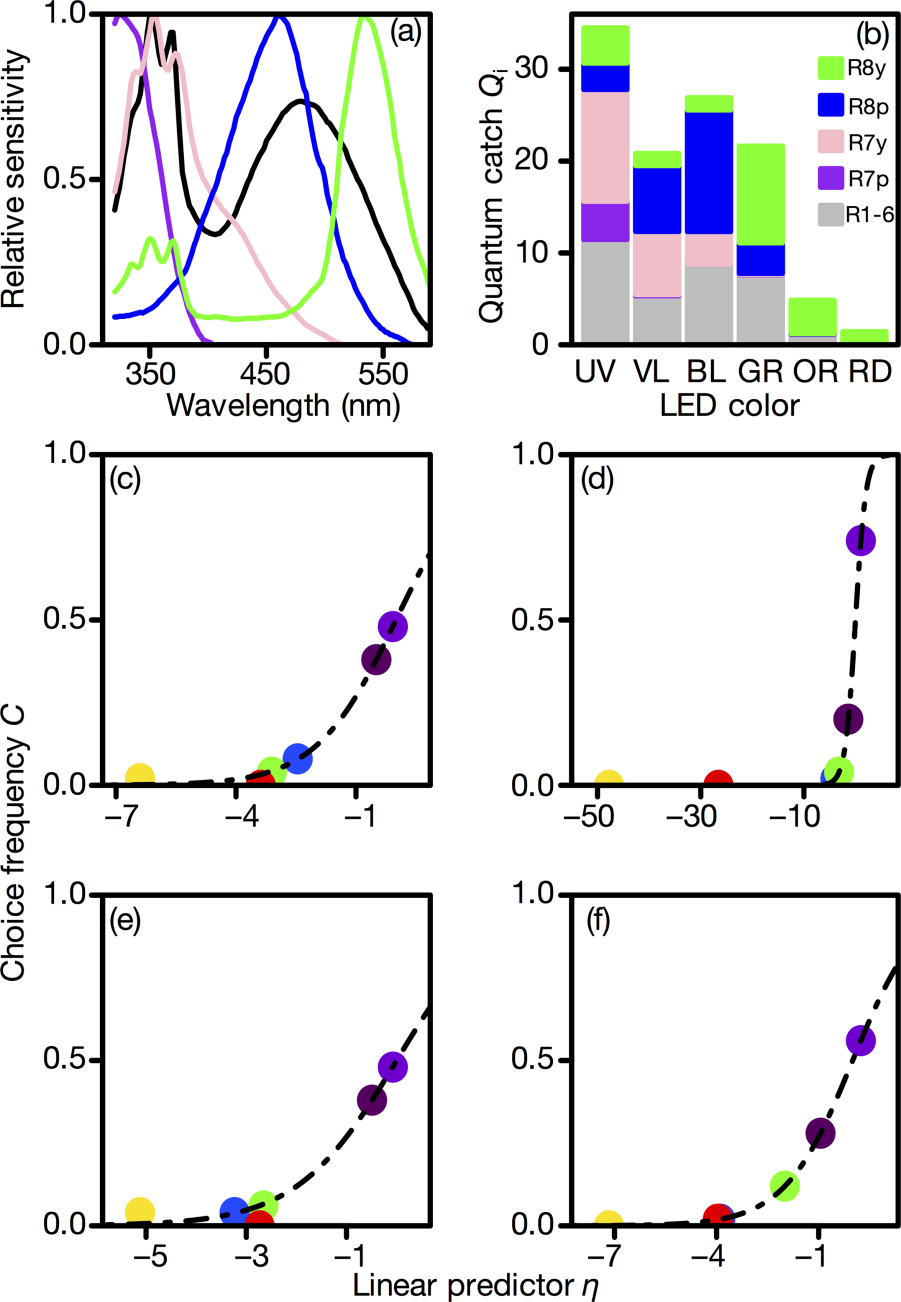
Photoreceptor-based modeling of wavelength preference. (a) Spectral sensitivities of the *Musca domestica* photoreceptors ([11]; the numerical data were obtained from [28]). (b) The relative quantum catch of individual photoreceptors, *Q*_i_. (c-f) Linear predictor in the best photoreceptor-based model versus choice frequency in (c) unmated males, (d) mated males, (e) unmated females, and (f) mated females. Circles indicate the measured choice frequency, and the dashed line is the value fitted by the best model as a function of the linear predictor η. The coefficients are shown in Tables 2–5.

The relative quantum catch of each photoreceptor class was calculated based on the spectral sensitivities of the house fly, *Musca domestica* [27–30] (Fig 4A and 4B), because the photoreceptor spectral sensitivities of the *E*. *japonica* are not known. This is a reasonable assumption for the ERG-determined spectral sensitivity of *E*. *japonica* eye, which is essentially identical to those of brachyceran flies, including *M*. *domestica*. It turned out that R1-6 catch quanta from all LEDs except for RD; R7p and R7y catch quanta mainly from UV and VL; and R8p and R8y catch quanta of BL and GR, respectively.

Tables 2–5 summarize the results of the photoreceptor-based model. The “best models” were chosen using *AIC*. The best model included terms for all receptor types in unmated males, unmated females, and mated females (Tables 2, 4 and 5). In this study, the signs of *Q*_R1-6_ and *Q*_R7y_ were positive, while those of *Q*_R7p_, *Q*_R8p_, and *Q*_R8y_ were negative. In the second-best model, *Q*_R7y_ was excluded, and the signs of the other terms were the same as in the best model. The *AIC* values were larger for the other models than for these two models. On the other hand, the best model for mated males included all of the receptor terms except *Q*_R7y_, and the second-best model included all of them (Table 3). The signs of terms were the same as in the model of the other experimental groups. All of the regression coefficients were significantly different from zero, except for mated males (Tables 2–5). These best models accurately fit the choice frequency of each LED (Fig 4C–4F).

**Table 2:** Regression coefficients and *AIC* for each model in unmated males. *ΔAIC* was defined as *AIC* subtracted from the minimum *AIC*. The row was sorted according to *AIC*.

bR1-6	bR7p	bR7y	bR8p	bR8y	*AIC*	*ΔAIC*
6.61* (z = 4.22)	-12.7* (z = -4.40)	0.22* (z = 2.58)	-4.25* (z = -4.45)	-3.69* (z = -4.51)	32.9	0
7.71* (z = 3.89)	-14.1* (z = -3.83)		-4.80* (z = -3.95)	-4.29* (z = -4.13)	38.1	5.2
		0.28* (z = 5.26)	-0.14* (z = -4.39)	-0.88* (z = -5.83)	78.0	45.1
	-0.21 (z = -0.89)	0.34* (z = 3.94)	-0.18* (z = -3.24)	-0.83* (z = -5.37)	79.2	46.3
0.01 (z = 0.05)		0.27* (z = 3.15)	-0.15* (z = -3.03)	-0.89* (z = -4.98)	80.0	47.1
-0.23* (z = -2.87)	0.37* (z = 2.45)	0.30* (z = 3.59)		-0.80* (z = -4.79)	83.3	50.4
-0.26* (z = -3.52)		0.38* (z = 5.27)		-0.66* (z = -4.90)	87.3	54.4
0.32* (z = 4.74)			-0.23* (z = -5.53)	-0.88* (z = -5.61)	88.3	55.4
	0.50* (z = 3.42)	0.10 (z = 1.90)		-1.02* (z = -5.62)	91.4	58.5
	0.63* (z = 4.94)			-0.81* (z = -7.02)	93.2	60.3
	0.57* (z = 4.34)		-0.02 (z = -1.04)	-0.73* (z = -5.51)	94.1	61.2
-0.02 (z = -0.63)	0.65* (z = 5.02)			-0.75* (z = -5.20)	94.8	61.9
		0.18* (z = 3.89)		-0.84* (z = -5.90)	101.5	68.6
-1.44* (z = -4.20)	2.35* (z = 3.08)	0.35* (z = 4.01)	0.68* (z = 2.99)		115.9	83
			-0.05* (z = -2.27)	-0.38* (z = -6.33)	116.0	83.1
				-0.44* (z = -7.57)	119.5	86.6
0.04 (z = 1.16)				-0.52* (z = -5.58)	120.1	87.2
-0.53* (z = -6.32)		0.45* (z = 5.60)			125.06	92.16
-0.54* (z = -6.00)	0.14 (z = 0.93)	0.43* (z = 4.89)			126.2	93.3
-0.53* (z = -5.95)		0.45* (z = 5.60)	0.00 (z = 0.07)		127.1	94.2
-0.26* (z = -7.54)	0.59* (z = 5.01)				156.9	124
	-1.23* (z = -4.82)	0.48* (z = 4.80)	-0.43* (z = -5.45)		165.2	132.3
-0.14* (z = -7.10)					181.7	148.8
-0.11* (z = -3.27)			-0.05 (z = -1.40)		181.72	148.82
		0.04 (z = 1.61)	-0.18* (z = -5.62)		191.2	158.3
			-0.15* (z = -6.20)		191.8	158.9
	-0.01 (z = -0.17)		-0.15* (z = -5.92)		193.8	160.9
-1.13* (z = -4.08)	2.55* (z = 4.00)		0.64* (z = 3.44)		225.8	192.9
	0.23 (z = 1.66)	-0.12* (z = -2.97)			233.4	200.5
		-0.06* (z = -3.07)			234.2	201.3
	-0.12 (z = -1.81)				240.7	207.8
(Null model)					242.2	209.3

* *p* < 0.05 (*z*-test).

**Table 3:** Regression coefficients and *AIC* for each model in mated males. *ΔAIC* was defined as *AIC* subtracted from the minimum *AIC*. The row was sorted according to *AIC*.

*b*_R1-6_	*b*_R7p_	*b*_R7y_	*b*_R8p_	*b*_R8y_	*AIC*	Δ*AIC*
65.36* (z = 5.28)	-121.32* (z = -5.28)		-40.07* (z = -5.27)	-34.47* (z = -5.33)	20.6	0
55.83 (z = 0.00)	-103.9 (z = -0.00)	10.29 (z = 0.00)	-34.30 (z = -0.00)	-29.48 (z = -0.00)	22.6	2
	-1.37* (z = -4.29)	0.73* (z = 6.15)	-0.36* (z = -4.06)	-0.91* (z = -5.28)	80.7	60.1
-0.46* (z = -3.86)		0.63* (z = 5.94)		-0.94* (z = -4.84)	88.2	67.6
-0.46* (z = -4.21)	-0.22 (z = -1.25)	0.66* (z = 6.36)		-0.84* (z = -4.42)	88.6	68
-0.51* (z = -3.56)		0.64* (z = 6.08)	0.03 (z = 0.60)	-0.88* (z = -4.22)	89.8	69.2
		0.40* (z = 5.45)	-0.11* (z = -3.50)	-1.42* (z = -6.19)	101.9	81.3
		0.30* (z = 4.71)		-1.36* (z = -6.21)	115.1	94.5
	0.05 (z = 0.33)	0.31* (z = 4.60)		-1.39* (z = -5.70)	117.0	96.4
-1.54* (z = -4.27)	1.37 (z = 1.64)	0.74* (z = 6.11)	0.56* (z = 2.23)		119.9	99.3
-1.02* (z = -7.02)		0.81* (z = 6.00)	0.15* (z = 2.70)		120.9	100.3
-0.90* (z = -4.90)	-0.39* (z = -2.03)	0.87* (z = 5.66)			123.3	102.7
-1.15* (z = -5.62)		0.99* (z = 5.36)			125.3	104.7
0.21* (z = 3.04)			-0.13* (z = -3.22)	-0.83* (z = -5.41)	140.2	119.6
	0.34* (z = 2.62)			-0.71* (z = -6.77)	142.6	122
0.00 (z = 0.21)	0.33* (z = 2.48)			-0.73* (z = -5.29)	144.5	123.9
	0.32* (z = 2.39)		-0.01 (z = -0.25)	-0.69* (z = -5.52)	144.5	123.9
				-0.54* (z = -7.92)	147.5	126.9
0.03 (z = -0.92)				-0.61* (z = -5.65)	148.6	128
			-0.02 (z = -0.93)	-0.51* (z = -6.79)	148.6	128
	-2.80* (z = -6.61)	1.02* (z = 5.81)	-0.77* (z = -5.04)		155.8	135.2
-0.97* (z = -4.08)	1.96* (z = 3.55)		0.56* (z = 3.49)		184.2	163.6
-0.21* (z = -7.03)	0.27* (z = 2.28)				211.7	191.1
-0.17 (z = -7.82)					214.7	194.1
-0.22* (z = -5.20)			0.06 (z = 1.41)		214.7	194.1
	-0.24* (z = -2.80)		-0.12* (z = -5.21)		244.9	224.3
			-0.14* (z = -5.83)		252.2	231.6
		0.01 (z = 0.32)	-0.14* (z = -4.86)		254.1	233.5
	-0.34* (z = -3.94)				277.0	256.4
	-0.32* (z = -2.25)	0.00 (z = -0.11)			279.0	258.4
		-0.07 (z = -3.63)			282.2	261.6
(Null model)					294.5	273.9

* *p* < 0.05 (*z*-test).

**Table 4:** Regression coefficients and *AIC* for each model in unmated females. *ΔAIC* was defined as *AIC* subtracted from the minimum *AIC*. The row was sorted according to *AIC*.

*b*_R1-6_	*b*_R7p_	*b*_R7y_	*b*_R8p_	*b*_R8y_	*AIC*	Δ*AIC*
5.39* (z = 4.90)	-10.7* (z = -5.23)	0.32* (z = 3.09)	-3.60* (z = -5.32)	-2.98* (z = -5.22)	37.4	0
5.92* (z = 4.66)	-10.8* (z = -4.59)		-3.73* (z = -4.797)	-3.30* (z = -5.00)	47.7	10.3
	-0.48 (z = -1.87)	0.37* (z = 3.90)	-0.25* (z = -3.73)	-0.59* (z = -5.25)	88.6	51.2
		0.22* (z = 4.93)	-0.16* (z = -4.70)	-0.65* (z = -5.63)	90.4	53
-0.07 (z = -0.61)		0.26* (z = 2.94)	-0.14* (z = -2.94)	-0.61* (z = -4.63)	92.0	54.6
-0.30* (z = -3.43)	0.31* (z = 2.15)	0.30* (z = 3.45)		-0.53* (z = -4.28)	96.2	58.8
-0.32* (z = -3.92)		0.38* (z = 5.00)		-0.46* (z = -4.19)	98.8	61.4
0.25* (z = 4.41)			-0.22* (z = -5.45)	-0.66* (z = -5.60)	99.2	61.8
	0.43* (z = 3.85)		-0.06* (z = -2.36)	-0.54* (z = -5.40)	104.8	67.4
-0.08 (z = -1.90)	0.60* (z = 4.80)			-0.54* (z = -4.88)	107.0	69.6
	0.51* (z = 4.33)			-0.68* (z = -6.70)	108.8	71.4
	0.45* (z = 3.18)	0.03 (z = 0.60)		-0.72* (z = -5.65)	110.4	73
		0.12* (z = 3.05)		-0.63* (z = -5.77)	118.8	81.4
			-0.07* (z = -3.00)	-0.32* (z = -6.04)	119.6	82.2
-0.54* (z = -6.24)		0.46* (z = 5.53)			120.4	83
-0.87* (z = -3.51)	1.01 (z = 1.71)	0.37* (z = 3.89)	0.25 (z = 1.47)		120.4	83
-0.57* (z = -5.80)	0.18 (z = 1.24)	0.43* (z = 4.73)			120.8	83.4
-0.53* (z = -5.58)		0.46* (z = 5.29)	-0.03 (z = -0.74)		121.8	84.4
				-0.40* (z = -7.42)	127.8	90.4
0.01 (z = 0.20)				-0.42* (z = -5.24)	129.7	92.3
-0.77* (z = -4.05)	1.74* (z = 3.94)		0.39* (z = 2.97)		136.9	99.5
	-1.52* (z = -4.92)	0.61* (z = 4.84)	-0.55* (z = -5.21)		148.8	111.4
-0.27* (z = -7.63)	0.62* (z = 5.16)				151.0	113.6
-0.09* (z = -2.90)			-0.08* (z = -1.99)		175.5	138.1
-0.15 (z = -7.21)					177.68	140.28
		0.05 (z = 1.88)	-0.21 (z = -5.88)		181.3	143.9
			-0.17* (z = -6.43)		182.9	145.5
	0.000 (z = -0.00)		-0.17 (z = -6.17)		184.9	147.5
	0.27 (z = 1.90)	-0.13* (z = -3.21)			230.1	192.7
		-0.07* (z = -3.20)			232.0	194.6
	-0.13(z = -1.81)				239	201.6
(Null model)					240.7	203.3

* *p* < 0.05 (*z*-test).

**Table 5:** Regression coefficients and *AIC* for each model in mated females. *ΔAIC* was defined as *AIC* subtracted from the minimum *AIC*. The row was sorted according to *AIC*.

*b*_R1-6_	*b*_R7p_	*b*_R7y_	*b*_R8p_	*b*_R8y_	*AIC*	Δ*AIC*
8.19*(z = 3.93)	-16.3*(z = -4.26)	0.43*(z = 3.28)	-5.39*(z = -4.24)	-4.36*(z = -3.99)	26.1	0
14.3*(z = 2.45)	-26.5*(z = -2.45)		-8.82*(z = -2.48)	-7.60*(z = -2.50)	40.9	14.8
	-0.90*(z = -3.52)	0.41*(z = 4.40)	-0.29*(z = -4.22)	-0.37*(z = -4.69)	111.4	85.3
-0.19(z = -1.65)		0.25*(z = 2.93)	-0.07(z = -1.43)	-0.35(z = -3.50)	122.8	96.7
-0.31*(z = -3.90)		0.30*(z = 4.23)		-0.29*(z = -3.27)	122.9	96.8
		0.12*(z = 3.46)	-0.12*(z = -4.09)	-0.45*(z = -5.38)	123.6	97.5
-0.31*(z = -3.87)	0.01(z = 0.08)	0.30*(z = 3.68)		-0.29*(z = -3.20)	124.9	98.8
0.12*(z = 2.57)			-0.14*(z = -3.90)	-0.45*(z = -5.20)	130	103.9
-0.49*(z = -6.38)		0.34*(z = 5.27)			133.4	107.3
	0.16(z = 1.68)		-0.07*(z = -2.87)	-0.36*(z = -5.26)	133.8	107.7
			-0.07*(z = -3.00)	-0.30*(z = -5.95)	134.7	108.6
-0.48*(z = -6.44)	-0.08(z = -0.51)	0.40*(z = 5.16)			135.2	109.1
-0.49*(z = -5.93)		0.39*(z = 5.28)	0.01(z = 0.25)		135.4	109.3
-0.41*(z = -3.09)	-0.33(z = 0.78)	0.44(z = 4.416)	-0.08(z = 0.53)		136.8	110.7
-0.08*(z = -2.26)	0.34*(z = 2.84)			-0.35*(z = -4.43)	137.5	111.4
	0.2(z = 1.93)			-0.46*(z = -6.17)	141	114.9
		0.05(z = 1.50)		-0.45*(z = -5.68)	142.3	116.2
				-0.38*(z = -7.30)	142.8	116.7
	0.17(z = 1.20)	0.01(z = 0.27)		-0.47*(z = -5.60)	142.9	116.8
-0.03(z = -1.05)				-0.32*(z = -4.75)	143.6	117.5
	-1.77*(z = -5.74)	0.65*(z = 5.28)	-0.56*(z = -5.32)		147.9	121.8
-0.47*(z = -4.29)	0.94*(z = 3.58)		0.20*(z = 2.50)		160.8	134.7
-0.22*(z = -7.25)	0.41(z = 3.55)				166.7	140.6
-0.16*(z = -7.50)					177.3	151.2
-0.13*(z = -3.80)			-0.03(z = -0.96)		178.4	152.3
	-0.12(z = -1.54)		-0.15*(z = -5.88)		193	166.9
			-0.16*(z = -6.30)		193.5	167.4
		0.02 (z = 0.72)	-0.18*(z = -5.38)		194	167.9
		-0.08*(z = -0.08)			233.3	207.2
	0.05*(z = 0.327)	-0.09*(z = -2.36)			235.2	209.1
	-0.24*(z = -3.08)				239	212.9
(null model)					247.1	221.0

* *p* < 0.05 (*z*-test).

## Discussion

Most insects including flies prefer UV [2] or GR [7, 35], which is likely related to the detection of the sky or plants [10, 36]. We unexpectedly discovered that the parasitoid fly preferred VL. To our knowledge, VL preference is rare in insects, and this is the first case in Diptera. More than half of the tested individuals preferred VL, irrespective of sex or mating experience. The preference for VL was particularly strong in mated flies. Another insect species that prefers VL is the predatory stinkbug, *Orius sauteri* [22]. In this bug, all individuals preferred VL (405 nm), except for mated females, who changed their preference to UV light (365 nm). Note that both *O*. *sauteri* and *E*. *japonica* are carnivorous insects that feed on insects. In herbivorous insects, green might be essential for landing and foraging on foliage [10]. Perhaps the VL preference is somehow related to host-searching or predatory behavior.

The preference for VL might be due to so-called wavelength-specific behavior, which is elicited by a specific photoreceptor class, with the maximal quantum catch for this particular wavelength. However, unlike certain butterflies [37], fly compound eyes do not contain narrow-band VL receptors, and therefore the VL preference cannot be attributed to a particular photoreceptor (Fig 2). Instead, we assume that color discrimination is involved in VL preference. Our model calculations suggest that signals from all photoreceptor classes, including the broadband R1-6 peripheral photoreceptors, contribute to VL preference. The classical theory is that the spectrally heterogeneous central R7 and R8 are the photoreceptors that deliver information for the color vision mechanism [12]. The concept, however, seems to be based on the process of adjustment. *Drosophila* uses all photoreceptor types for innate and acquired wavelength preferences [38], and the R1-6 photoreceptors are essential for wavelength discrimination [39]. Our results are consistent with these recent studies. In this study, we performed choice tests, using monochromatic lights, with a certain equal quantum flux. Studies evaluating lights of varying intensity would provide further support of our hypothesis.

The statistical modeling suggested a subtractive interaction among the spectral types of photoreceptor. In the best model, the regression coefficients of R1-6 and R7y were positive, and those of R7p, R8p, and R8y were negative. However, the coefficient of R7y was 0 or < 1 (Table 2–5). This suggests that the contribution of R7y to VL preference is considerably smaller than those of the other receptors. In addition, the subtractive interaction indicates that spectral opponency, *i*.*e*., antagonism among the signals from the photoreceptors, is implemented, as suggested in *Drosophila* [3, 40]. There are neurons that are excited by some photoreceptors and inhibited, directly or indirectly, by other photoreceptors [41]. The broadband sensitivity of R1-6 suggests that they are suitable for discriminating light intensities. It is theoretically possible to reduce redundancy in the signals of overlapping spectral sensitivities by subtracting the signals of narrowband receptors from those of broadband receptors [42]. This process probably enables the flies to extract heterogeneity in the wavelength distribution of light.

The natural enemies, including the parasitoid flies, are regarded as alternatives to pesticides in IPM. There have been attempts to attract and retain natural enemies in agricultural fields, but no effective methods have yet been established for parasitoid flies. Because many insects are attracted to UV or GR, VL is a promising candidate for selectively attracting *E*. *japonica* to the agricultural field [18]. Additional behavioral experiments in the field are necessary to exploit the unique nature of this species for controlling lepidopteran pests.

## Supporting Information

S1 Dataset Irradiance spectra of the LEDs. (CSV)

S2 Dataset Number of flies attracted to each LED. (CSV)

S3 Dataset Spectral sensitivity of compound eye in *Exorista japonica*. (CSV)
